# Longitudinal fluorescent observation of retinal degeneration and regeneration in zebrafish using fundus lens imaging

**Published:** 2013-05-23

**Authors:** Michèle G. Duval, Helen Chung, Ordan J. Lehmann, W. Ted Allison

**Affiliations:** 1 Department of Biological Sciences, University of Alberta, Edmonton Alberta, Canada; 2 Department of Ophthalmology, University of Alberta, Edmonton Alberta, Canada; 3 Department of Medical Genetics, University of Alberta, Edmonton Alberta, Canada; 4 Centre for Prions and Protein Folding Diseases, University of Alberta, Edmonton Alberta, Canada

## Abstract

**Purpose:**

Longitudinal observation of retinal degeneration and regeneration in animal models is time-consuming and expensive. To address this challenge, we used a custom fundus lens and zebrafish transgenic lines with cell-specific fluorescent reporters to document the state of individual retinal neurons in vivo.

**Methods:**

We empirically tested several versions of a custom fundus lens and assessed its capabilities under a stereomicroscope to image retinal neurons in transgenic zebrafish lines expressing fluorescent reporters. Vascular branch points provided spatial references enabling determination of whether changes induced by ablating photoreceptors were repaired over the course of several days.

**Results:**

Individual ultraviolet- and blue-sensitive cone photoreceptors were readily visualized in vivo, and green fluorescent protein–labeled blood vessels were used as landmarks to facilitate orientation. Sequential imaging of the same retinal areas over several weeks permitted documentation of photoreceptor reappearance in individual animals. Photoreceptor regeneration in these regions was evidenced by the reappearance of individual fluorescent cells.

**Conclusions:**

This technique permits real-time in vivo serial examination of individual fish, permitting temporal analysis of changes to the retinal mosaic. The key benefits this technique offers include that the same retinal locations can be recovered and viewed at multiple time points, that in vivo observations are comparable to those made ex vivo, and that fewer animals need to be euthanized over the course of an experiment. Our results promise the ability to detect individual cells, including reappearing cone photoreceptors, and to monitor disease progression during screening of therapies in an adult animal model of late onset disease.

## Introduction

Interventions to influence retinal degeneration and repair benefit from longitudinal study, in which one can assess tissue- and cellular-level changes in retinal morphology or function. Thus, rather than measuring the degree of degeneration or the effects of therapeutic treatments at a single time point per individual animal euthanized, assays are more informative and ethical if each animal can provide meaningful data at multiple time points. Examples of such technologies in fish models of vision science include electroretinography [1-5] and behavioral measures such as the optokinetic response [6-8]. Other approaches have recently allowed visualization of the zebrafish retina in vivo using light microscopy [9], though these methods are not able to image individual fluorescing cells as we sought to achieve. Elegant techniques enabling repeated measures of morphology are emerging in animal models of ophthalmology, e.g., optical coherence tomography (OCT) [10], though the complexity and expense can be prohibitive.

Zebrafish have emerged as a major model system that effectively complements murine models. In ophthalmology, the similarities of the zebrafish and human retinas encompass structure, development, and function. Zebrafish have abundant cone photoreceptors, including multiple spectral subtypes [11-16], which provide an effective model of macular disease. Thus, zebrafish are particularly suitable for studying photoreceptor degeneration and regeneration [17-22]. The conservation of structure and function extends to the inner retina, permitting effective models of glaucomatous change [23,24] in ganglion cells and vasculature, as well as retinopathy associated with angiogenesis [25]. In addition, zebrafish possess a robust regenerative capacity, study of which promises to be influential in emerging stem cell and gene therapies to repair vision loss in humans [19-21]. This increasing utility is also driven by the growing list of mutant and transgenic lines available, and the increasing ease with which new mutants can be generated. An additional, oft celebrated, benefit is the transparency of zebrafish tissues and the accessibility this provides, although it is restricted to early development (about 5 days after fertilization) since larval and adult fish lose transparency as pigmentation develops.

Although genetic and pharmacological interventions are practical in zebrafish, and visual function can be measured with electroretinograms or studies of visual behavior [21,26], documenting changes in retinal morphology is more challenging. Traditionally, this has been limited to histology and labeling of fixed tissues, although OCT technology has recently been adapted to empower zebrafish researchers examining retinal degeneration [10]. Ideally, one would be able to image the retina en face, providing the same view as obtained with an ophthalmoscope, and in this report, we describe the development and testing of an economical fundus lens that can be used to longitudinally visualize fluorescent cells in all layers of the zebrafish retina. With this fundus lens in the optical path of a fluorescent stereomicroscope (equipment available in most institutions and zebrafish research groups), individual cells and vasculature can be visualized in transgenic animals expressing fluorescent reporters. We demonstrate resolution of single cells, including discerning spatial patterns among neighboring cells. Using endogenous and experimentally-induced landmarks in the retina, this method allows visualization of the same retinal location over multiple time points. This technology allows documentation of changes in cell abundance or alterations in gene expression compatible with longitudinal study design, by visualizing retinal cells in vivo.

## Methods

### Animal husbandry

Zebrafish were maintained under standard rearing conditions at the University of Alberta. Lighting was provided by fluorescent bulbs on a 14 h:10 h light-dark cycle. Transgenic animals used in this study included those expressing green fluorescent protein (GFP) or its derivatives Kaede or mCherry in subsets of retinal cells (Table 1). These cells included retinal ganglion cells, bipolar cells, cone photoreceptors with or without fluorescent vasculature (n=9 tested; images and video from seven shown), and vasculature alone. Lectin peanut agglutinin conjugated to Alexa Fluor 568 (Invitrogen # L32458; Burlington ON, Canada) was delivered to the intraocular space via a fine gauge needle in anaesthetized fish. All procedures adhered to the Association for Research in Vision and Ophthalmology Animal Statement and were approved by the University of Alberta Animal Care and Use Committee under the auspices of the Canadian Council on Animal Care.

**Table 1:** Cell types visualized and transgenic fish expressing fluorescent reporters in cognate cells.

**Retinal Cell Layer**	**Cell type visualized**	**Transgenic line**	**ZFIN ID^a^**	**Source [Reference]**
Ganglion Cell Layer	Retinal Ganglion cell	*Tg(−17.6isl2b:GFP)zc7*	ZDB-GENO-100322–8	Chien Lab [34]
Inner Nuclear Layer	Vasculature	*Tg(fli1a:EGFP)y1*	ZDB-GENO-011017–4	Weinstein Lab [35]
	Bipolar cells^b^	*Et(fos:Gal4-VP16)s1181t; Tg(UAS-E1b:Kaede)s1999t*	ZDB-ALT-080327–149; ZDB-GENO-070314–3	Baier Lab / ZIRC [29]
	Müller Glia	*Tg(gfap:GFP)mi2001*	ZDB-GENO-060623–2	Raymond Lab [36]
Outer Nuclear Layer	UV-sensitive cones	*Tg(−5.5opn1sw1: EGFP)kj9*	ZDB-GENE-991109–25	Kawamura Lab [37]
	Blue-sensitive cones	*Tg(−3.5opn1sw2: mCherry)ua3011*		Allison laboratory [22]; promoter from Kawamura [38]
	Rods	*Tg(−3.7rho:EGFP)kj2*	ZDB-GENO-060830–2	Kawamura Lab [39]

^a^ZFIN ID refers to the unique name assigned to this transgenic line at the Zebrafish Model Organism Database, zfin.org and the associated Zebrafish International Resource Centre. ^b^We putatively assign these cells as bipolar cells near the optic nerve head based on morphology, see Figure 8; Appendix 4.

### Positioning of custom lens and image acquisition

**Figure 1:**
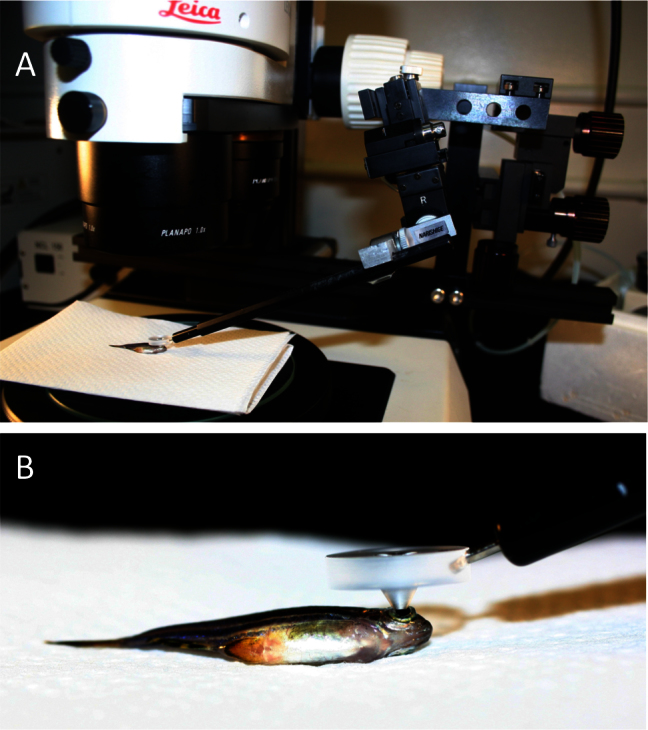
Mounting of a custom fundus lens on a fluorescent stereomicroscope allows characterization of individual photoreceptor cells in vivo. **A**: An adult anaesthetized zebrafish is shown, positioned on its flank under the objective lens such that its pupil is in the centre of the field-of-view. The fundus lens is positioned in the light path, centered above and touching the pupil (detailed in **B**). A micromanipulator (right side of image) allows precise and constant positioning of the fundus lens but is not required. **B**: A view of the custom fundus lens in position for viewing, positioned on the fish eye. The ventral side of the fish is in view.

Adult zebrafish were anaesthetized in MS-222 and positioned under a fluorescent stereomicroscope (Leica MZ16F with EL6000 mercury metal halide fluorescent light source; Concord ON, Canada) such that the pupil of one eye was fully visible and in focus. To facilitate identifying the same location in multiple trials, fish were positioned with the ventral (inferior) side toward the observer. Several iterations of custom plastic fundus lenses produced by Ocular Instruments (Bellevue, WA) were empirically tested, with lens P03221A providing the optimal combination of magnification and large field of view. This lens, catalog number P03221A, is now commercially available from Ocular Instruments. The fundus lens was positioned on the corneal surface so that the lens lay in the light path between the microscope objective and the zebrafish pupil (Figure 1). Coupling fluid was tested (Tear-Gel; Novartis, Mississauga, Canada), and was found neither a hindrance nor a necessity for visualization, so the fluid was not used during collection of the results presented here. Although we found the fundus lens practical as a handheld instrument for routine use, use of a micromanipulator was advantageous for imaging and training purposes. One of two mechanical micromanipulators was employed: an economical two-axis design (MM33; Sutter Instrument, Novato, CA) or a three-axis model (MMN-1; Narshige, East Meadow, NY) that enhanced control of the fundus lens position (in three dimensions). All images and video presented were obtained with the latter micromanipulator.

Imaging commenced at low magnification microscope settings and the pupil positioned in the center of the field of view. The fundus lens was applied to the cornea and the vertical position adjusted until the fluorescent light emitted from the retinal cells came into focus. Increasing magnification and refinement of focus were then primarily driven by the microscope controls. Navigating to more peripheral retina was achieved by lateral repositioning of the fundus lens relative to the cornea, which rotated the globe and moved different areas of the retina into the light path. Still images and movies were collected using a 12.8 megapixel digital camera (DP72, Olympus; Richmond Hill ON, Canada) mounted on the stereomicroscope. Images were manipulated for brightness and contrast in ImageJ64 for Apple Mac OSX (Wayne Rasband, National Institutes of Health), and the movies were edited and annotated in iMovie HD 5.0.2 (2005, Apple Computer Inc., Cupertino CA).

### Photoreceptor ablation via light damage

We used previously described methods of photoablation [17,27] to study subsequent photoreceptor regeneration; the toxic light dose induces death in photoreceptors, perhaps via excitotoxicity or accumulation of heat, but leaves other retinal cells intact. Adult fish were placed in a 200 mL Pyrex beaker containing 100 mL of fish tank water with a mercury metal halide fluorescent light source (Leica EL6000) positioned at the perimeter of the beaker at a level illuminating the area where the fish could swim. A similar method was deployed previously to study photoreceptor regeneration [28], and ablation of photoreceptors in our experiment similarly occurred along a horizontal streak across the nasal-temporal axis of the eye. The fish were exposed to this toxic light for 10 min before being returned to normal husbandry conditions. Retinal lesions were visualized at 1, 4, 8 (alternatively at 7), 10, 28, and 35 days post-lesion (DPL); the time points of the images are indicated in the appropriate figures.

### Retinal flatmounting and histology

To visualize retinas ex vivo, fish were dark-adapted overnight, given a lethal dose of MS-222, and the cervical spinal cord severed. Retinas were dissected away from other ocular tissues and either flatmounted for immediate imaging or fixed in 4% paraformaldehyde if imaging was deferred to a later time.

The expression of fluorescent reporters in various retinal cell types is well established in the majority of transgenic lines deployed (Table 1), except in one instance that permitted testing the ability to image cells in the inner nuclear layer. This line, *Et(fos:Gal4-VP16)s1181t/+;Tg(UAS-E1b:Kaede)s1999t/+*, was generated during an enhancer trap screen and thus has Gal4 expression under control of an unknown endogenous zebrafish promoter region. Gal4 binds the 14XUAS promoter to drive expression of the fluorescent reporter Kaede. This line has been annotated regarding Kaede expression in larval zebrafish [29], especially including expression in Mauthner cells of the hindbrain, but not in adults or retinas. After the in vivo imaging, the fish was euthanized, and the eyes were fixed in paraformaldehyde and prepared for visualization by cryosectioning. TO-PRO-3 (Invitrogen) was added to visualize the nuclei, and the sections were visualized using confocal microscopy (Zeiss LSM 700 on Axio Observer.Z1; Toronto ON, Canada).

## Results

### Viewing cone photoreceptors and identifying landmarks in vivo

**Figure 2:**
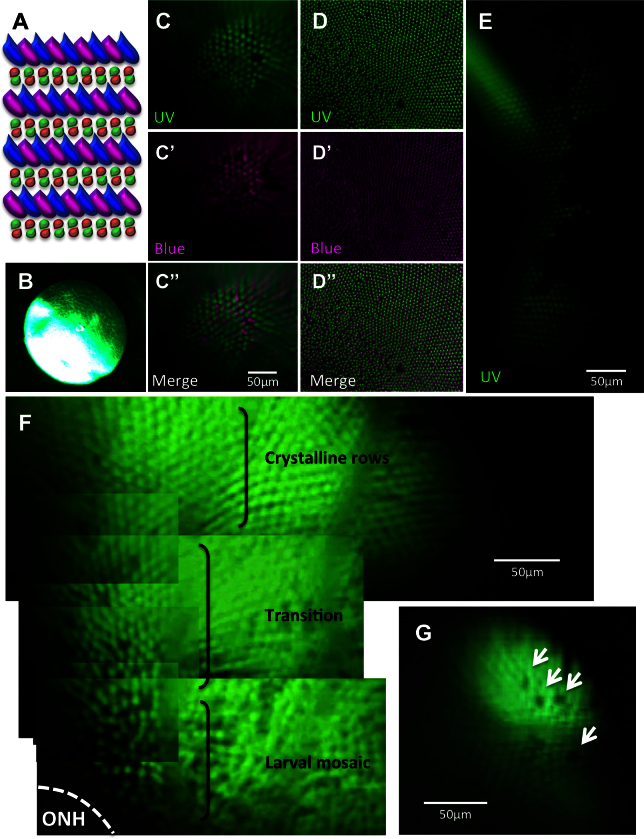
Viewing the cone mosaic in vivo. **A**: The cone row mosaic is shown as a schematic (with ultraviolet-sensitive (UV) cones in magenta and blue-, green- and red- sensitive cones coloured as per their respective spectral sensitivity; not to scale). B. The best possible picture equivalent to C acquired without the fundus lens, using standard stereomicroscope optics. C. The cone mosaic can be successfully imaged in vivo; The example here is from fish transgenic for green fluorescent protein (GFP; **C**) and mCherry [**C**’] in UV- and blue-sensitive cones, respectively, and merging the two channels [**C**”] with mCherry signals pseudocoloured to magenta. **D**: An ex vivo image of a flat-mounted retina dissected away from other ocular tissues is presented for comparison to [**C**, **D**], [**D**’] and [**D**”] show GFP, mCherry and merged channels, respectively. **E**: Details are visible in vivo after cell ablation, such as lesion edges where GFP-labeled cones have died, leaving clear gaps in the cone mosaic. **F**: A panorama assembled from stills taken in Appendix 2 (0:08-0:12) depicts several features, including: the crystalline rows of UV cones in the adult row mosaic; the larval mosaic, which does not contain rows; and a transition zone between the mosaics. The optic nerve head (ONH) is located at the bottom left of the panorama. **G**: An image of UV cone rows with gaps, representing individual absent cones, which was regularly observed. Appendix 1 showing intact photoreceptors, and Appendix 2 of one intact and two lesioned photoreceptor layers, can be found online. n=4 fish shown in this image.

The cones of adult zebrafish are highly conserved with those of mammals in most respects, except that the zebrafish cone mosaic has remarkable precision regarding the arrangement of neighboring cone subtypes [14,15,30]. This is schematized in Figure 2A, summarizing that various methods from previous research [11-16] led us to expect ultraviolet (UV)- and blue-sensitive cones to occur in a precise alternating relationship within rows radiating from the central retina. This pattern is less regular near the optic nerve head [14]. With existing stereomicroscope optics, we cannot visualize small structures such as vessels or individual cells (Figure 2B). Thus, we challenged our fundus lens technology with the ability to resolve individual cone photoreceptor cells in vivo. We found that our imaging allowed us to discriminate individual UV- and blue-sensitive cone photoreceptors to a degree comparable with the stereomicroscope’s resolution of ex vivo tissue (Figure 2C,D, Appendix 1). The ability to discriminate individual cells was confirmed when images of UV- and blue-sensitive cones, containing GFP and mCherry, respectively, were merged. The expected alternating pattern of cone subtypes was apparent and comparable to ex vivo imaging (Figure 2C,D). Finally, we noted that detailed patterns of cones could be documented, including crystalline rows of each subtype, which could be merged to reveal the cone row mosaic (Figure 2C,F), a less precise arrangement of cones near the optic nerve head suspected of being a remnant of the larval mosaic and/or cell rearrangements [14,22], the transition between the two patterns (Figure 2F), innate gaps in the crystalline rows where a cone is absent or not expressing the transgene (Figure 2G), and edges of the lesions we had induced by light ablation (Figure 2E, Appendix 2). Based on comparisons to ex vivo images, the field of view using the fundus lens can range from 100 μm to 270 μm with the capacity to resolve individual cell bodies (approximately 4 μm; these comparisons were required to estimate object size in images, because the new optical path, incorporating the fundus lens and the fish’s endogenous lens, obviated use of the microscope’s size calibration). Increased coverage of the retina for imaging is conveniently attained by montages of images or by video capture (e.g., Figure 2E, Appendix 1, Appendix 2).

Examination of more than 20 individual animals, not all of which are shown here, typically during multiple sessions and including the various transgenes described, suggests that the approach can work robustly in almost every individual fish. Exceptions to the latter were rare fish with occluded/cloudy lenses.

**Figure 3:**
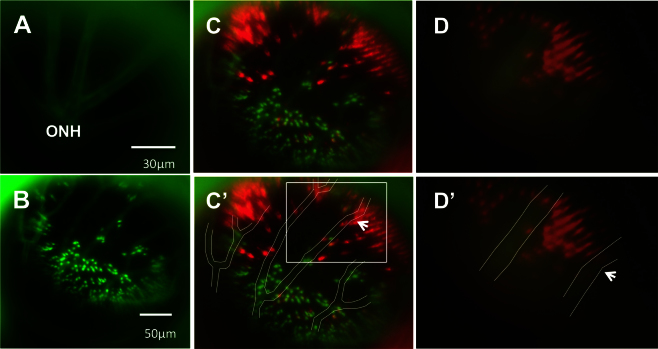
Using vasculature patterns as in vivo landmarks to navigate within the eye. **A**: An in vivo view of the optic nerve head (ONH) is represented by the apparent convergence of vessels. From the ONH, vessel branching patterns were utilized to record and relocate areas of interest. **B**: A low-magnification image of the green fluorescent protein (GFP) channel is presented, showing the branching pattern of vessels that is used to navigate during imaging. Due to the difference in focal planes of the vessels and photoreceptor layer, the apparent positions of individual photoreceptors may shift slightly relative to the vessels. **C**: The same image in **B** is presented with GFP and mCherry channels merged and vessels outlined in white for clarity [**C**’]. Due to discrepancies in focal plane between the photoreceptor layer and the vessels, relative positions of some small areas of interest and neighbouring vessels differ slightly between images and between magnifications. To illustrate this, an area of interest, a bay-shaped lesion edge, was imaged at different magnifications. In **C**’, the area of interest is located between two vessel branches (white box). **D**: The area, imaged at high magnification, is shown without [**D**] and with vessels outlined [**D**’]. At high magnification, the area lies underneath one of the vessel branches (arrows indicate the same branch in each image), an apparent a shift of approximately 57 μm. n=2 fish shown.

To facilitate longitudinal study, retinal landmarks were characterized to permit imaging of the same retinal locations in individual fish at multiple time points. The optic nerve head, entirely lacking photoreceptors, was readily identifiable (Figure 2F). The outline of blood vessels and the movement of blood within vessels was also apparent (Appendix 1, 0:38–0:47; Appendix 2, 0:00–0:14); however, these views of blood vessels were impaired when the fluorescing cells behind the blood vessels had been ablated. To facilitate use of blood vessels as landmarks, the transgenic lines were crossed such that GFP was expressed in the vasculature and the UV-sensitive cones, and mCherry in blue-sensitive cones. The GFP signals from cones and vasculature, occurring in different layers of the retina, were readily separable based on the plane of focus (Figure 3).

Areas of interest were mapped relative to the optic nerve head (ONH), seen as an apparent convergence of vessels (Figure 3A) and/or absence of photoreceptors (Figure 2F). The branching of blood vessels after they exit the optic nerve head was easy to document, allowing us to confidently identify retinal locations despite the lack of fluorescent signals from the cones following photoreceptor ablation (Figure 3B–D). The ONH was used as a center-point in navigating the retina during separate viewing sessions. Once the ONH was located, the branching patterns of the vessels were recorded (e.g., in video) and used as guides to repeatedly recover the various locations previously viewed.

**Figure 4:**
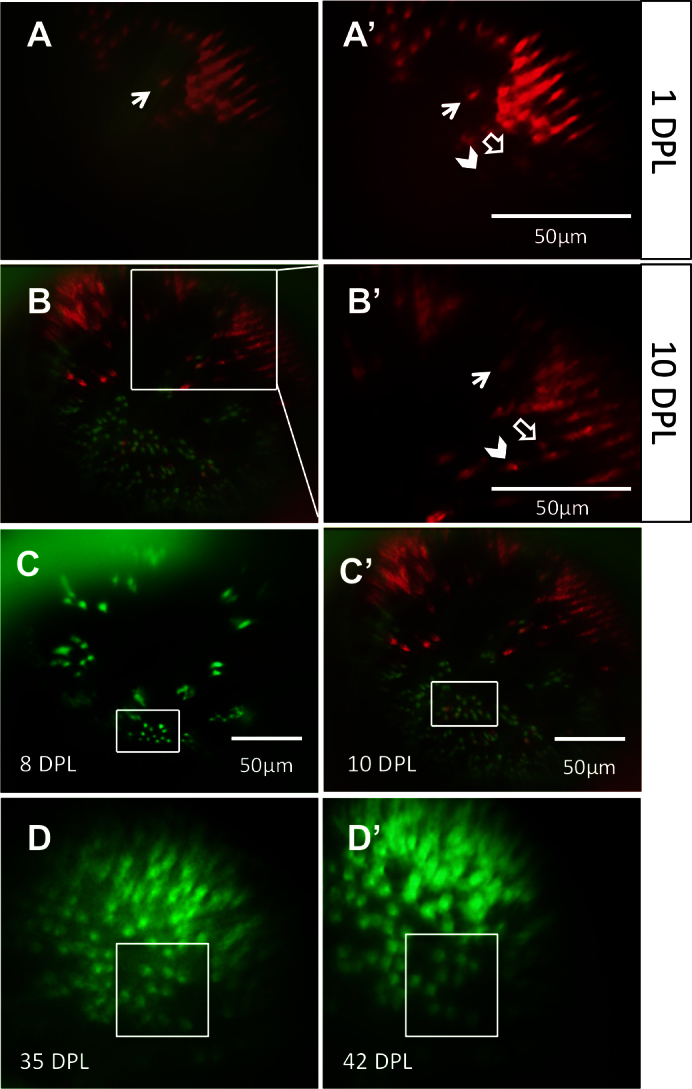
The same locations on the retina can be recovered and viewed on subsequent days. Through a combination of approximate relocation using vessel branch patterns and more precise relocation using unique lesion edge patterns, areas of interest were reexamined at multiple time points. **A**: In the mCherry channel (blue-sensitive cones), a lesion edge reminiscent of a bay shape, and a single cone within the bay (arrow), was observed at 1 day post-lesion (DPL). Image in A is a merge of the green fluorescent protein (GFP) and mCherry channels; **A**’ shows the mCherry channel alone. **B**: The same bay-shaped lesion edge was relocated at 10 DPL. **B** is a merge of the GFP and mCherry channels; B’ shows a higher magnification of the white box in **B**, in the mCherry channel alone. The single cone (arrow) was also identified. The appearance of a new blue-sensitive cone between 1 DPL [**A**’] and 10 DPL [**B**’] is indicated with an empty arrow. The chevron indicates a potential surviving cone, but the faint signal from this location at 1 DPL puts to question if the cone died and was replaced by the bright cone seen at 10 DPL. **C**: An area containing uniquely-shaped patches of surviving ultraviolet-sensitive cones is observed at 8 DPL (top) and relocated at 10 DPL (bottom). The same collection of ultraviolet-sensitive cones, and the same individual cones, was identified (white boxes) at both days. **D**: An area of regenerated UV cones showing the loss of row mosaic organization, imaged at 35 and 42 DPL (panel **D** and **D**’, respectively); matching clusters of cones are indicated with white boxes. For an example of the outlined vessels and how areas were located using vasculature, see Figure 3. n=2 fish shown.

**Figure 5:**
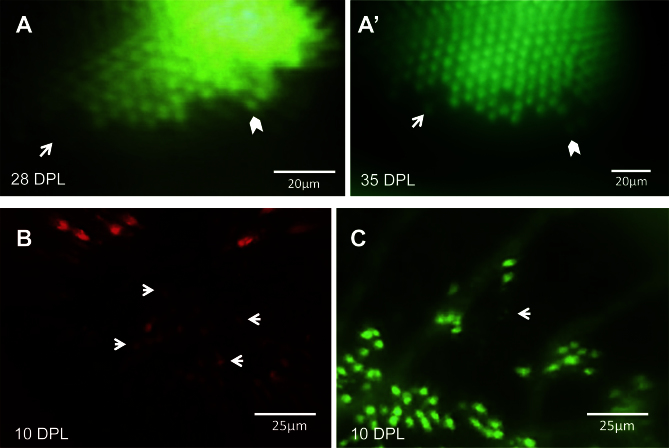
Changes in the retina over time can be captured in the same eye. **A**: An unltraviolet-sensitive (UV) cone that regenerated between 28 days post-lesion (DPL) [**A**] and 35 DPL [**A**’] is indicated with white arrows. White chevrons indicate the same cone in both panels, used to match the areas on different days. **B**: Faint spots of fluorescence (some examples shown with arrows) suggest that new blue-sensitive cones are beginning to express sws2 opsin (mCherry) at 10 DPL, and appear smaller and fainter than the surviving cones seen at the lesion edge in upper half of image. **C**: The same retina as shown in **B**, in the GFP channel; the white arrow indicates one or two new UV-sensitive cones beginning to express sws1 opsin. Faint GFP expression of new UV cones, alongside cones with robust expression, can be viewed in Appendix 3. n=2 fish shown.

Because the blood vessels and the photoreceptor layer are in different focal planes, the perceived location of small areas of interest varies slightly relative to the vasculature when the magnification is changed. For example, when a large field of view is examined, a location such as the bay-shaped area of interest in Figure 4 and Figure 5 appears between two vessel branches. However, when the magnification is increased, the area appears to shift directly underneath one of the vessel branches (Figure 3). Variation in relative positions of objects is minimal (approximately 40–50 microns) between magnifications and thus does not impinge on the utility of the vasculature as landmarks.

### Longitudinal study of changing cone abundance

**Figure 6:**
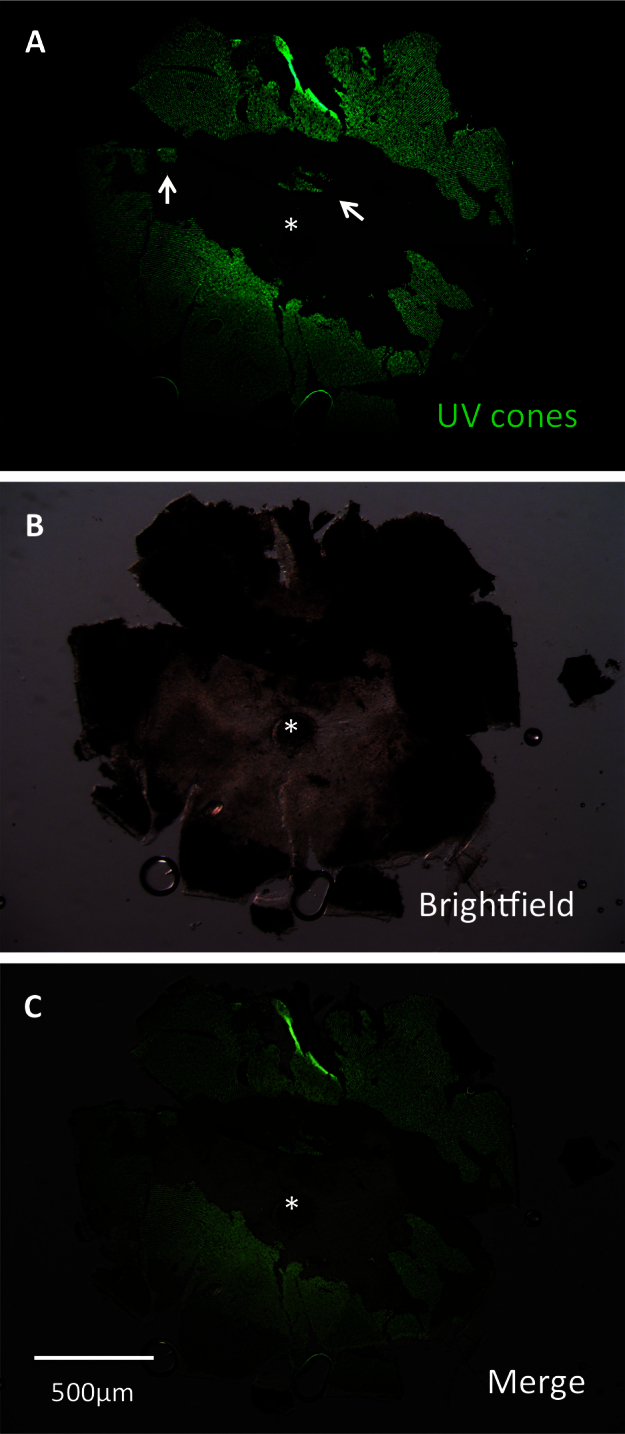
A lesioned retina was dissected to assess efficacy of lesioning methods. This retina was dissected at 3 days post-lesion (DPL) and flatmounted for imaging. Lesioning occurred across the eye. Lesion edges were observed to vary between clean, straight edges (center of retina) and variable shapes (left and right edges, representing periphery of retina). Patches of surviving cones (arrows) were commonly observed. **A**: The image is presented showing green fluorescent protein (GFP), which is contained in ultraviolet-sensitive (UV) cones. **B**: A brightfield view of the same retina. **C**: A merge of images **A** and **B** is presented. The asterisk indicates the optic nerve head (ONH).

Photoreceptors were ablated using exposure to high intensity light and examined in vivo. A subset of retinas was additionally examined ex vivo to assess lesion size (Figure 6). Patches of surviving cones were observed within lesion areas (Figure 6; Appendix 2).

Following cone photoreceptor ablation, these vascular landmarks were used to return to the same retinal location. Using vascular branching patterns, unique to each fish and the retinal region around the optic nerve head, the same regions were readily identified in examinations several days apart (Figure 4). In many instances, this general location was then refined by identifying unique patterns of photoreceptors at the edge of the light-lesioned area, as demonstrated by identification of a distinct, bay-shaped lesion edge that was located first via vessel branch patterning and subsequently by the area’s unique shape (Figure 3 and Figure 4).

**Figure 7:**
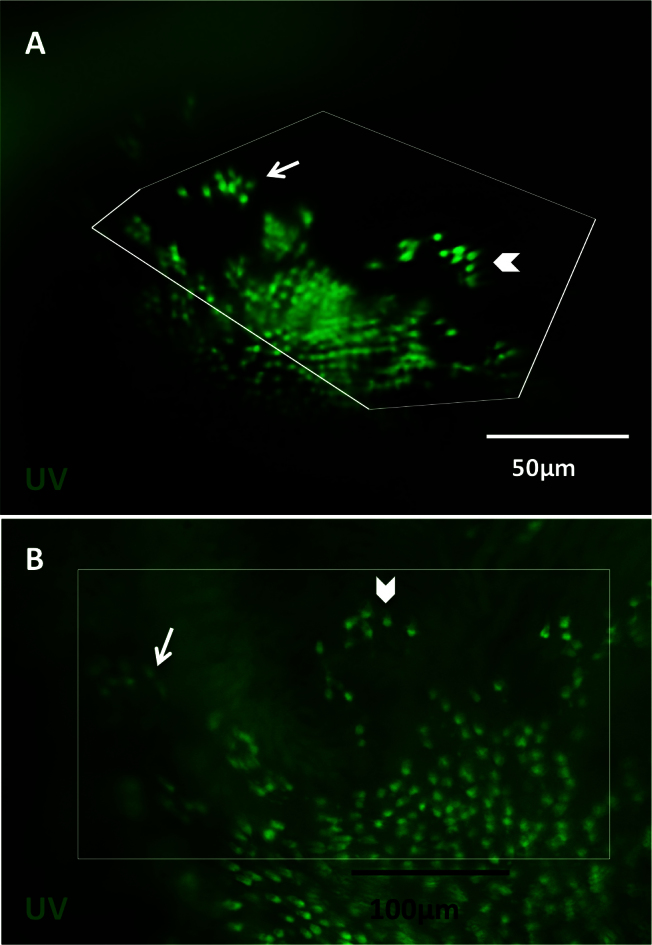
Observations made in vivo using the fundus lens are comparable to those made ex vivo. Images were captured in vivo at 10 days post-lesion (DPL), followed by sacrifice the same day to compare imaging results. In vivo images obtained with the fundus lens [**A**] were compared to the flatmounted retina dissected away from other ocular tissues [**B**]. Images **A** and **B** show ultraviolet (UV)-sensitive cones in the green fluorescent protein (GFP) channel. White box represent corresponding areas between in vivo and ex vivo; the distortion of the boxes seen in vivo is a result of flattening the curvature of the eye for best visualization during dissection. Arrows and chevrons indicate matching clusters of UV cones. Blood vessels were removed during dissection, so are not visible ex vivo. Background fluorescence is due to preservation of the dissected retina in fixative before mounting.

Within lesion areas where no fluorescent signal was detected 1 day following ablation, faint signals were found 7–10 days following ablation, which were assumed to represent new cones (Figure 4 and Figure 5, Appendix 3). This interpretation is based on the signals’ appearance in lesion patterns between viewings, but also due to the considerably fainter GFP or mCherry expression, seen best in Appendix 3 (0:11–0:14), where faint punctate signals occur adjacent to bright, healthy cones regardless of focal plane adjustment. These cells were interpreted as being newly regenerated, in as few as 7 days, from the endogenous stem cells shown to be activated by this light ablation method in past experiments, though cell fate markers such as BrdU were not used here to confirm the source [28]. Comparison of in vivo images of newly appearing cones with those obtained ex vivo (dissected from the eye following sacrifice of the fish) revealed that the former produced images with serviceable quality and resolution (Figure 7), validating that the in vivo lens technique faithfully represented photoreceptor distributions.

### Visualizing other cell types

In addition to cone photoreceptors and vasculature, the ability to visualize other retinal cell types was explored. Fluorescence of Müller glia and rods was apparent in their respective transgenic lines, although visualizing individual rods or Müller glia was unsuccessful in these preliminary experiments (data not shown).

**Figure 8:**
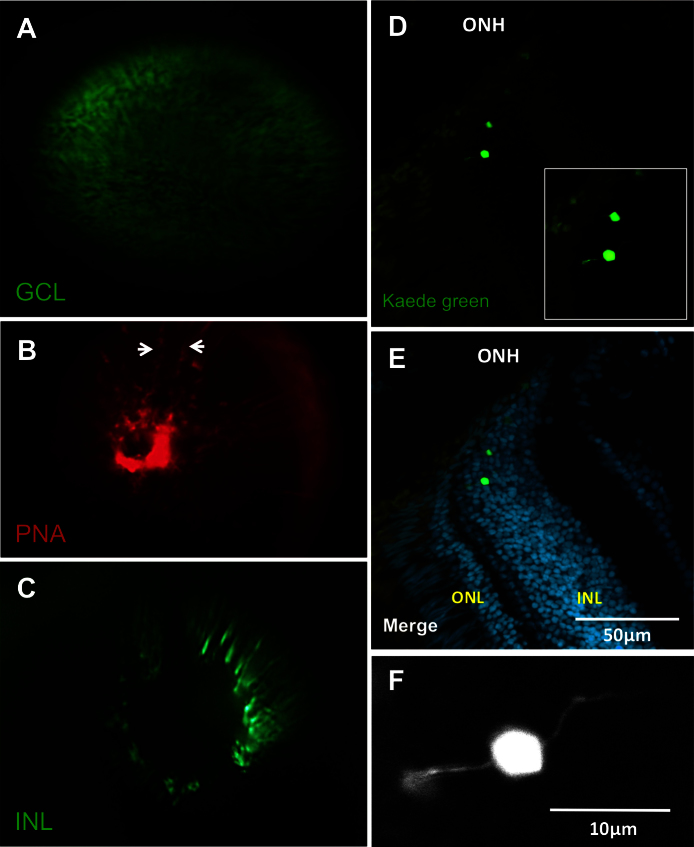
Each of the three nuclear layers viewed under the fundus lens. **A**: View of a transgenic fish eye with green fluorescent protein (GFP) in the retinal ganglion cells. **B**: The vessels of the inner eye partially labeled with lectin peanut agglutinin (PNA; arrows indicate edges of a vessel) as an alternative to using the fli1a:EGFP line. **C**: View of putative bipolar cells of the INL in a fish of the *Et(fos:Gal4-VP16)s1181t; Tg(UAS-E1b:Kaede)s1999t* line. See Appendix 4 to view this line in vivo. **D**: Example cryosection of the eye visualized in C, chosen to show bipolar cell morphology near the optic nerve head. **E**: Same as **D**, with nuclear stain. The location of the Kaede-labeled cells was confirmed to be the INL, near the ONH. **F**: Higher magnification view of inset in image **D**, showing bipolar morphology. Apical and basal extensions to left and right are consistent with bipolar cell morphology. ONH: optic nerve head; ONL: outer nuclear layer; INL: inner nuclear layer. Video of the INL can be found online in Appendix 4. n=3 fish shown.

Examination of other lines of transgenic fish permitted visualization of individual ganglion cells (Figure 8A). Individual cells of the INL were also visualized, clustered around the optic nerve head (ONH) of an enhancer trap line (Figure 8C). These cells were putatively identified as bipolar cells based on the INL position and morphology in histological sections of tissue adjacent to the ONH (Figure 8D–F). Lectin peanut agglutinin was applied in an attempt to label cone photoreceptors [31] in fish that lack transgenes (Figure 8B). The cones were not labeled in a discernible fashion using intraocular delivery of lectin peanut agglutinin (as demonstrated with ex vivo visualization of cones in mice following in vivo lectin delivery [31]); however, the fluorescent signal was abundant surrounding the vasculature, suggesting this could be an alternative approach to the transgenes used above.

## Discussion

We described a method that enables repeated measures of fluorescent reporters and cell distributions in the adult zebrafish neural retina. The method enables longitudinal study of cell degeneration and regeneration that will serve as an effective complement to existing longitudinal measures of retinal function. Longitudinal measures enable study of changes within an individual that can provide greater interpretive power, lower husbandry cost, and decrease the number of animals that are euthanized. The approach expands upon existing technology that is limited to light microscopy [9], thus substantially increasing the types of individual cells that can be studied. Recent work demonstrated the utility of OCT in zebrafish degeneration work [10], though this approach is thus far also limited in which cell types can be identified. The method reported here is economical, simple to implement, and expands the optical capabilities of microscopy infrastructure that is nearly ubiquitous in zebrafish research groups.

Overall, the results demonstrate that individual cells of transgenic fish can be imaged in vivo to an unprecedented level of resolution comparable to the stereomicroscope’s ability to image the same tissue dissected from the eye. Further, the method enables examination of the same retina during trials separated by multiple days. The technique employed endogenous and experimentally induced landmarks in the retina, allowing us to repeatedly document the same photoreceptors following light-induced cell ablation. Previous work has shown that intense light exposure leads to ablation of photoreceptors in fish [17,27,28], and can be used to study degeneration or subsequent regeneration of cells. Intriguingly, this methodology documented the appearance of new fluorescent objects in the retina 7 days following ablation (Figure 4, Figure 5), and we interpreted these objects as regenerating photoreceptor cells. The possibility that these newly appearing photoreceptors were undetectable at earlier time points cannot be excluded. However, it is well established that photoreceptor ablation induces stem cell proliferation and subsequent regeneration over a similar time course to these studies [28]; thus, the majority of these reappearing cells likely represent regenerated cones. Accordingly, this fundus contact lens may provide a novel capability for serially imaging this regenerative process.

Our past work quantified cone photoreceptor patterns and their relative abundance in retinas dissected from adult zebrafish [14,22]. The images obtained provide in vivo confirmation of two observations made during that study: that the mosaic pattern of cones is less precise near the optic nerve head (denoted as the larval remnant mosaic remodeled throughout normal adulthood [14,22]; Figure 2F) and that some GFP-expressing UV cones are missing from the precise array of cones in vivo (Figure 2G, Appendix 2). The data presented here show that these characteristics exist in living fish, eliminating our hypotheses that these characteristics were artifacts of the past dissection procedures. Indeed, these characteristics are bona fide and should be integrated into interpretations of data assessing photoreceptor distributions in zebrafish.

The fundus lens facilitated visualization of cells in all retinal layers. This included resolving individual retinal ganglion cell bodies. Because ganglion cells are progressively lost in glaucoma, this technology may be applied to repeatedly measure the effects of treatments in zebrafish models of glaucoma. We also visualized the cells of the inner nuclear layer. The transgene expression in the particular line of zebrafish used is driven by random insertion of a reporter in the genome [29], such that the cell-type specificity of expression is not predictable. Our characterization of this transgenic line suggests that fluorescence is contained in a subset of bipolar cells (Figure 8D–F, Appendix 4). Bipolar cell regeneration has been a recent topic of interest, being made accessible by new chemically inducible transgene technology in zebrafish [32]. The fundus lens presented here might enable additional insights into bipolar cell regeneration. Finally, the fundus lens did not resolve individual rod photoreceptors or Müller glia in the transgenic lines we investigated. This is likely due to the density of the cells and the intense brightness of their fluorescent signals prohibiting resolution of individual cells. This result does not preclude use of the fundus lens with these transgenic fish where the brightness of the signal would be an informative experimental outcome. For example, a loss of total rod opsin protein during photoreceptor degeneration can be a useful outcome, and longitudinal studies of inflammation associated with neurodegeneration often use abundance of glial fibrillary acidic protein abundance as an informative outcome for assessing treatment efficacy. Alternatively, one can envisage study of these cells using transgenic lines that deploy other labeling technologies or where genetically mosaic animals are available, though we did not pursue this line of research in this study.

Regarding attempts to establish useful retinal landmarks, the fundus lens is especially effective in visualizing retinal vasculature and blood flow. This ability has significant potential to enable longitudinal study of retinopathy and macular degeneration, including recent hypoxia-induced models of angiogenesis [25] using this same transgenic line. The capacity to robustly visualize vasculature and blood flow in repeated measures could also be informative in defining the time course of angiogenesis associated with tumorigenesis. This could in some instances be facilitated by delivery of fluorescently labeled lectin peanut agglutinin, because it was able to selectively label some cell types, including vasculature. The delivery of other dyes, such as recently described coumarin dyes [33], might allow additional cell types to be resolved, whereas deploying GFP reporters of transcriptional activity could permit a unique combination of spatial and temporal information. Finally, this technology should be amenable to other species, including but not limited to mouse and *Xenopus*, with accessible transgenesis technology.

In conclusion, we have engineered an economic and straightforward addition to the standard zebrafish microscopy suite that enables visualization of fluorescent reporters relevant to ophthalmological disease processes. The ability to resolve such reporters to the level of individual cells repeatedly in vivo will empower longitudinal investigations of treatments and processes in animal models of retinal disease progression and repair.

## Appendix 1.

Video supplement to Figure 2. In vivo view of the intact cone photoreceptor layer; UV cones are labeled in GFP. The crystalline structure of the adult cone row mosaic, the larval mosaic, and occasional gaps in the row mosaic are included. Streaks where cones are out of focus are caused by vessels obscuring fluorescence. n=2 fish shown. To access the data, click or select the words “Appendix 1.”

## Appendix 2.

Video supplement to Figure 2. Views of an intact retina (including larval remnant and optic nerve head, readily identified by a gap in fluorescence) and lesion edges in two zebrafish 1 day post-lesion. Both fish are transgenics expressing GFP and mCherry in UV and blue cones, respectively. To access the data, click or select the words “Appendix 2.”

## Appendix 3.

Video supplement to Figure 5. View of lesion edges in a transgenic zebrafish 7 days post-lesion; newly appearing UV cones are visible as faint spots below the lesion edge, which are interpreted as being regenerated cones. The fish is a transgenic expressing GFP and mCherry in UV and blue cones, respectively. To access the data, click or select the words “Appendix 3.”

## Appendix 4.

Video supplement to Figure 8. View of GFP-labeled cells of the inner nuclear layer, potentially bipolar cells, surrounding the optic nerve head, in a fish of the *Et(fos:Gal4-VP16)s1181t;Tg(UAS-E1b:Kaede)s1999t* transgenic line. To access the data, click or select the words “Appendix 4.”
